# Imprinting of the Polycomb Group Gene *MEDEA* Serves as a Ploidy Sensor in Arabidopsis

**DOI:** 10.1371/journal.pgen.1000663

**Published:** 2009-09-25

**Authors:** Aleksandra Erilova, Lynette Brownfield, Vivien Exner, Marisa Rosa, David Twell, Ortrun Mittelsten Scheid, Lars Hennig, Claudia Köhler

**Affiliations:** 1 Department of Biology and Zurich-Basel Plant Science Center, Swiss Federal Institute of Technology, Zurich, Switzerland; 2 Department of Biology, University of Leicester, Leicester, United Kingdom; 3 Gregor Mendel Institute of Molecular Plant Biology, Austrian Academy of Sciences, Vienna, Austria; The University of North Carolina at Chapel Hill, United States of America

## Abstract

Balanced maternal and paternal genome contributions are a requirement for successful seed development. Unbalanced contributions often cause seed abortion, a phenomenon that has been termed “triploid block.” Misregulation of imprinted regulatory genes has been proposed to be the underlying cause for abnormalities in growth and structure of the endosperm in seeds with deviating parental contributions. We identified a mutant forming unreduced pollen that enabled us to investigate direct effects of unbalanced parental genome contributions on seed development and to reveal the underlying molecular mechanism of dosage sensitivity. We provide evidence that parent-of-origin–specific expression of the Polycomb group (PcG) gene *MEDEA* is causally responsible for seed developmental aberrations in Arabidopsis seeds with increased paternal genome contributions. We propose that imprinted expression of PcG genes is an evolutionary conserved mechanism to balance parental genome contributions in embryo nourishing tissues.

## Author Summary

Crosses between plants of different ploidy often fail because seed development does not proceed normally and non-viable seeds are produced. It is assumed that abnormalities in growth and structure of the endosperm (the nutritional tissue of the seed) are the cause of triploid seed failure, consistent with the proposed role of the endosperm in reproductive isolation and angiosperm speciation. In many species, the ratio of maternal to paternal genomes in the endosperm is important for normal seed development, giving rise to the hypothesis that parent-of-origin–specific gene expression (imprinting) of regulatory genes in the endosperm is the underlying cause for developmental failure in seeds with deviating parental contributions. We tested this hypothesis using the *jason* mutant that forms unreduced male gametes and triploid seeds with increased paternal genome dosage. Based on the results of our study, we propose that imprinting of the FIS component *MEDEA* serves as a dosage sensor for increasing paternal genome contributions, establishing the molecular basis for dosage sensitivity. Our study provides strong evidence supporting the hypothesis that misbalanced expression of imprinted genes is the cause of seed development defects after interploidy crosses and demonstrates that *MEDEA* imprinting is a major origin of developmental defects caused by increased paternal genome contributions.

## Introduction

Polyploidy, the presence of more than two complete sets of chromosomes within an organism, is known to be common in plants and in some animals such as amphibians, fish and reptiles [1],[2]. The widespread occurrence of polyploids among plant species suggests that polyploidy is evolutionary beneficial and represents a major mechanism for plant adaptation and speciation [2]–[6]. The additional sets of chromosomes may originate from the same species (“autopolyploid”), or from the hybridization of two different species (“allopolyploid”). Polyploids can arise spontaneously by the fusion of a diploid gamete with a normal haploid gamete, leading to the formation of a triploid zygote. Diploid gamete formation resulting from failure of reduction during meiosis occurs in several plant species and can give rise to triploids that serve as a bridge to the formation of stable polyploids with an even set of chromosomes [4].

In most flowering plants the fusion of one sperm cell with the haploid egg cell is accompanied by the fusion of a second sperm cell with the homodiploid central cell nucleus, resulting in the formation of the triploid endosperm with a 2∶1 ratio of maternal to paternal genomes. The endosperm is a nourishing tissue supporting embryo growth [7]. Double fertilization occurs also in polyploids, resulting in the formation of embryo and endosperm with proportionally increased ploidies. However, crosses between plants of different ploidy often fail because seed development does not proceed normally and non-viable seeds are produced, a phenomenon that has been termed “triploid block” [8]. It is assumed that abnormalities in growth and structure of the endosperm are the cause of triploid seed failure [9], consistent with the proposed role of the endosperm in reproductive isolation and angiosperm speciation [10]–[12]. In many species the 2∶1 ratio of maternal to paternal genomes in the endosperm is required for normal seed development [12],[13], giving rise to the hypothesis that gene dosage effects and imprinting of regulatory genes in the endosperm is the underlying cause for developmental failure in seeds with deviating parental contributions [9],[14],[15]. Genomic imprinting is the mitotically stable inheritance of differential expression states of maternal and paternal alleles caused by different epigenetic modifications of the alleles. Genomic imprinting renders maternal and paternal genomes non-equivalent, and balanced contributions of maternal and paternal genomes are therefore essential for post-fertilization development [15]–[17].

The penetrance of the interploidy hybridization barrier varies within and between species. Whereas maize is particularly sensitive to changes in the ratio of maternal to paternal genome contributions in the endosperm [13],[14], in *Arabidopsis thaliana* a substantial accession-dependent variation in the degree of postzygotic seed lethality has been observed in crosses between individuals of different ploidy [18],[19]. The phenotype of viable seeds resulting from reciprocal interploidy crosses is different, depending on which parent contributes the higher genome dose. Whereas seeds developing from a cross of a diploid female with a tetraploid male develop larger endosperm, the reverse is true when the maternal plant contributes the double genome dose [19]. Increased endosperm growth in seeds with increased paternal genome contribution is associated with an increased number of mitotic divisions and delayed cellularization [19]. This phenotype is strikingly similar to the endosperm phenotype of Arabidopsis *fertilization independent seed* (*fis*) mutants that are characterized by an uncellularized endosperm with increased number of nuclei [20],[21]. The FIS genes *MEDEA* (*MEA*), *FERTILIZATION INDEPENDENT ENDOSPERM* (*FIE*), *FIS2* and *MSI1* encode components of an evolutionary conserved Polycomb group (PcG) complex that deposits repressive chromatin modifications on a defined set of target genes [21]–[26]. The FIS PcG complex has been implicated in the regulation of imprinted genes [27], and the FIS components *MEA* and *FIS2* are themselves regulated by genomic imprinting with only the maternal alleles being expressed [28]–[30].

In this work, we addressed the question whether seeds with increased paternal genome contribution have reduced FIS PcG activity causing developmental aberrations in the endosperm. We identified a novel mutant forming unreduced pollen that enabled us to investigate direct effects of unbalanced parental genome contributions on seed development. We show that parent-of-origin specific expression of *MEA* is causally responsible for seed developmental aberrations in triploid Arabidopsis seeds. Seeds containing triploid embryos and tetraploid endosperm (referred to as “triploid seeds”) are characterized by increased expression of FIS PcG target genes during late stages of seed development and increased transgene induced *MEA* expression in the endosperm can significantly suppress developmental defects of triploid seeds. Our findings reveal the underlying molecular mechanism of dosage sensitivity and suggest that imprinted expression of PcG genes is an evolutionary conserved mechanism to balance parental genome contributions in embryo nourishing tissues.

## Results

### The *jason* Mutant Forms Enlarged Triploid Seeds with Increased *PHE1* Expression

**Figure 1:**
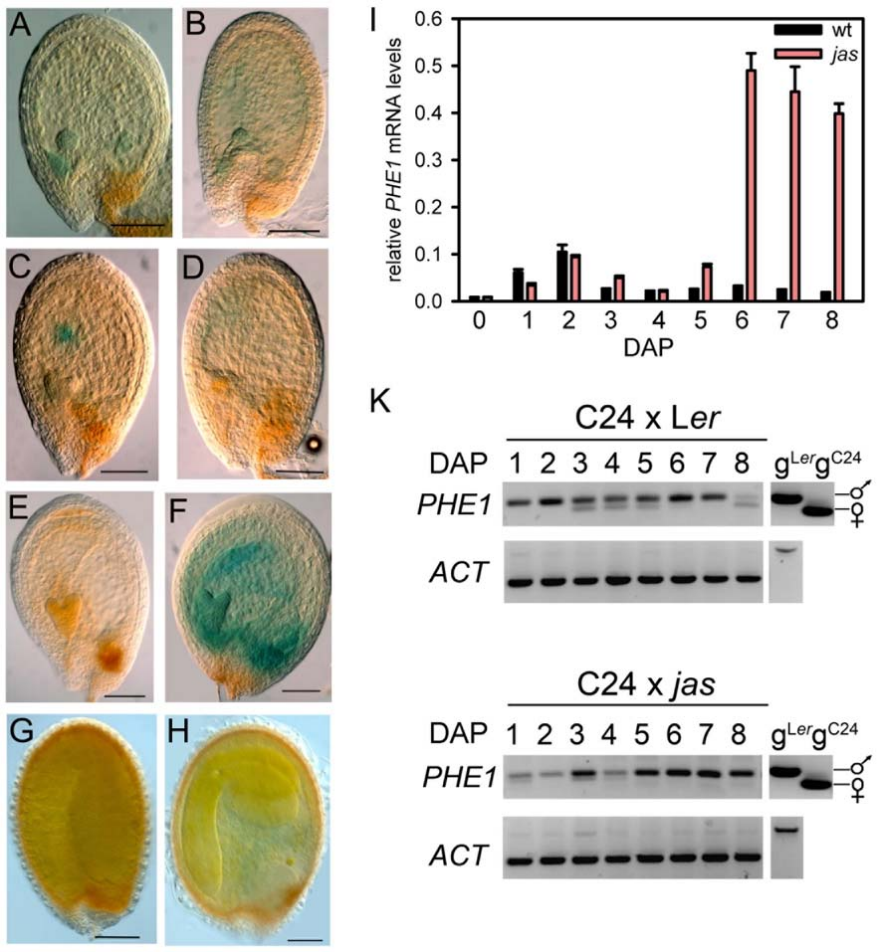
Overexpression of *PHE1::GUS* and the endogenous paternal *PHE1* allele in seeds of *jas* plants. (A,C,E,G) *PHE1::GUS* expression in wild-type seeds, (B,D,F,H) *PHE1::GUS* expression in seeds of selfed homozygous *jas* plants. Seeds were analyzed at 4 (A,B), 5 (C,D), 6 (E,F) and 10 DAP (G,H). Scale bars, 100 µm. (I) Quantitative RT–PCR analysis of *PHE1* expression in wild-type and *jas* seeds. Error bars, s.e.m. (K) *PHE1* imprinting is not affected in *jas* seeds. Allele specific *PHE1* transcript levels were determined after crosses of C24 with wild-type (L*er*) or *jas* plants [27]. DAP; days after pollination. wt; wild type.

*PHERES1* (*PHE1*) is a direct target gene of the *Arabidopsis thaliana* FIS PcG complex and is predominantly expressed in the endosperm during early stages of seed development [31]. *PHE1* expression ceases around the time of endosperm cellularization, when the embryo has reached late heart stage. We performed a genetic screen aimed at identifying novel regulators of *PHE1* expression and made use of a previously established reporter line containing the *PHE1* promoter fused to the *β-GLUCURONIDASE* (*GUS*) reporter [31]. The identified *jason* (*jas-1*) mutant had strongly increased GUS staining in the endosperm starting at 5 to 6 days after pollination (DAP), when embryos had reached late heart stage, whereas the GUS staining pattern was indistinguishable from the wild type during early seed development (Figure 1A–1H). Increased GUS staining was restricted to the endosperm, apparent staining of the embryo is a consequence of the endosperm overlaying the embryo. We tested whether increased expression levels of the reporter gene were reflected by increased expression of the endogenous *PHE1* gene and analyzed *PHE1* expression at defined DAP (Figure 1I). Consistent with the results from the *PHE1* reporter construct, increased endogenous *PHE1* expression was observed from 5 DAP onwards.

*PHE1* is an imprinted gene that is predominantly paternally expressed [27]. Therefore, we investigated whether increased expression of *PHE1* in *jas* mutant seeds is caused by breakdown of *PHE1* imprinting. We crossed the Arabidopsis accession C24 as female with pollen from Landsberg *erecta* (L*er*) wild type or *jas* mutant and analyzed allele-specific expression of *PHE1* in seeds resulting from these crosses. However, no increase in maternally derived *PHE1* transcripts was detected in *jas* mutant seeds and increased *PHE1* transcript levels were solely established by increased expression of the paternal *PHE1* allele (Figure 1K).

**Figure 2:**
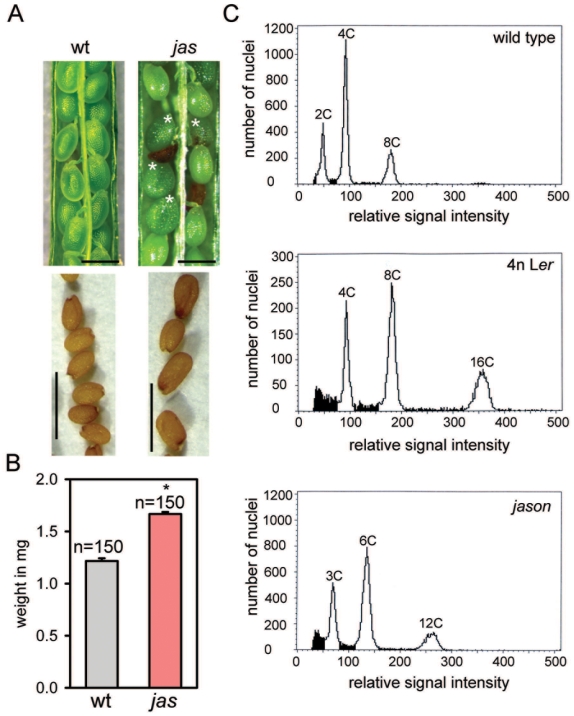
Homozygous *jas* plants produce significantly larger triploid seeds. (A) Silique from wild-type and *jas* plants after self-fertilization. Triploid *jas* seeds are enlarged (white asterisks) and sometimes abort (dark brown seeds). Lower panels show ripe seeds. Scale bars, 0.5 mm upper panels, 1 mm lower panels. (B) Seed weight of wild-type and *jas* plants. Average seed weight is given in mg per 50 seeds. Significance was determined by two-tailed Student's t-test, *P<0.01. Numbers above bars indicate numbers of scored seeds per genotype. Error bars, s.e.m. (C) Representative flow cytometry histogram plots of nuclei from wild-type, tetraploid (4n) and seedlings grown from enlarged *jas* seeds.

Development of *jas* seeds is delayed compared to wild-type seeds: whereas wild-type seeds reached maturity at 10 DAP, *jas* seeds reached maturity only after about 18 DAP (Figure 1G and 1H). Mature *jas* seeds were significantly increased in size (Figure 2A and 2B), resembling seeds derived from 2n×4n crosses [19]. Therefore, we tested whether the *jas* mutation caused triploid seed formation by measuring the ploidy levels of the progeny of diploid homozygous *jas* plants. Indeed, among the *jas* progeny we found diploid and triploid (45%) but no tetraploid seedlings (10 triploids among 22 plants; Figure 2C). Seedlings grown from size-selected enlarged *jas* seeds were all triploid, indicating that enlarged seeds are indeed triploid (n = 51). These data suggest that the *jas* mutation causes diploid gamete formation at high frequency, however, the presence of diploid seedlings as well as normal sized seeds among the progeny of *jas* plants (Table 1) indicates that the *jas* mutation is not completely penetrant.

**Table 1:** Seed phenotype of reciprocal crosses between *jas* and wild-type plants.

Female	L*er* (selfed)	*jas* (selfed)	*jas*	L*er*
	**	**	**×**	**×**
Male			L*er*	*jas*
Unfertilized	9%	11%	4%	10%
Normal	91%	51%	95%	42%
Enlarged	0%	36%	0%	45%
Aborted	0%	2%	1%	3%
n (seeds)	406	758	918	737

Green or dry siliques resulting from self pollination or out-crosses to wild-type L*er* plants were opened and the seeds classified as normal, enlarged, aborted, or unfertilized ovules. n (seeds), number of seeds scored.

The *jas* mutant is sporophytic recessive; mutant plants were detected at a ratio of about 25% among segregating F2 plants (n = 185; (χ^2^ = 0.134<χ^2^_0.05[1]_ = 3.84)), indicating normal transmission of the *jas* allele through male and female gametes. Also, JAS does not appear to have a general role in seed development, as abnormal seed development was not observed in seeds from selfed *jas* heterozygous plants, even though 25% of the seeds are homozygous for the *jas* allele. In order to test whether the *jas* mutation affects male or female gametogenesis, we reciprocally crossed *jas* plants with L*er* wild-type plants and analyzed seeds developing from these crosses. Enlarged seed formation was only observed when the *jas* mutation was paternally transmitted (Table 1 and Figure S1), suggesting that *jas* pollen is diploid. Conversely, we did not detect significant numbers of abnormally-sized seeds when the *jas* mutation was maternally transmitted (Figure S1), strongly suggesting that *jas* only affects male gametogenesis. Additional support for this conclusion was derived from the finding that all F1 seedlings derived from crosses of *jas* plants with wild-type pollen were diploid (n = 40). Together, triploid seed formation in the identified *jas* mutant is caused by a defect during male gametogenesis leading to unreduced gamete formation.

### The *jason* Mutant Forms Unreduced Male Gametes at High Frequency

**Figure 3:**
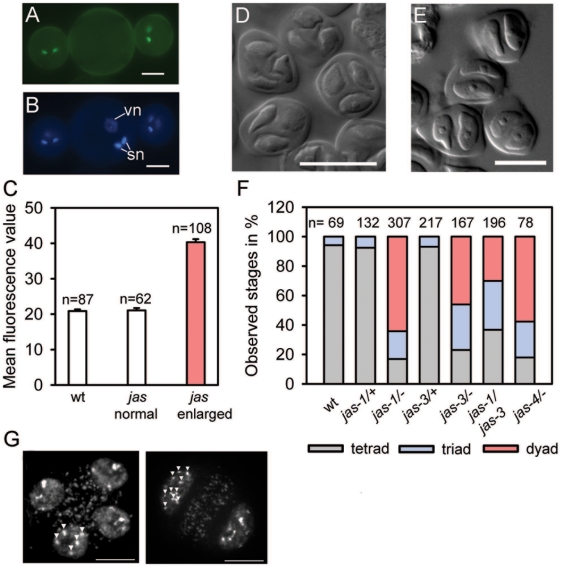
Homozygous *jas* plants form dyads and enlarged diploid pollen. (A) Pollen from *jas* plants was mixed with wild-type pollen marked by the sperm cell marker *MGH3::H2B-GFP* [65]. GFP negative enlarged pollen grain is derived from *jas* plants. Scale bar, 10 µm. (B) DAPI staining of pollen shown in panel (A). Enlarged *jas* pollen contains two sperm nuclei (sn) and one vegetative nucleus (vn). (C) DNA content of sperm cells in mature pollen from wild type and *jas* plants. Bars show mean relative DNA contents (DAPI fluorescence values) for pollen from wild type (WT) or *jas* plants, with *jas* pollen classified on the basis of size; normal sized (*jas*) or enlarged (*jas* enlarged). The mean fluorescence from the enlarged pollen was approximately twice that of the wild type and normal sized *jas* pollen, indicating that it has twice the DNA content. wt; wild type. (D) Tetrad formation in wild-type plants. Scale bar, 25 µm. (E) Dyad formation in *jas* plants. Scale bar, 25 µm. Scale bar, 10 µm. (F) Quantification of meiotic products in L*er* wild-type, *jas-1/+*, *jas-1/−*, *jas-3/+*, *jas-3/−*, *jas-1/jas-3* and *jas-4/−*. Numbers on top of columns indicate numbers of analyzed meiotic products. Triads observed in wild type are most likely caused by spore superposition. (G) Chromosomal spreads of wild-type tetrad (left panel) and *jas* dyad (right panel). Chromosomes are marked by arrow heads.

Diploid pollen is significantly larger than haploid pollen [32], and consistent with our hypothesis that *jas* pollen is diploid, we observed 62% enlarged pollen in *jas* plants (n = 144; Figure 3A and 3B). Furthermore we measured DNA content of sperm cells from wild-type and enlarged *jas* pollen. The mean fluorescence from the enlarged pollen was approximately twice that of the wild-type and of normal-sized *jas* pollen, indicating that the former has twice the DNA content and is thus diploid (Figure 3C). To define the stage of pollen formation affected by the *jas* mutation, we analyzed the meiotic products of *jas* microspore mother cells. Whereas wild-type plants almost exclusively formed tetrads, *jas* plants formed dyads and triads at high frequency (64% dyads, 19% triads, n = 307 Figure 3D–3F). Thus, *JAS* is required in the sporophyte to regulate male meiosis and a meiotic defect is responsible for the formation of diploid pollen in *jas*. Consistent with this view we frequently observed ten chromosomes in dyad microspores, in contrast to the five chromosomes observed in tetrad microspores (Figure 3G and data not shown). The subsequent mitotic divisions are not affected by the *jas* mutation; all pollen grains formed by *jas* mutant plants contained two sperm cells and one vegetative cell (Figure 3B).

To define the meiotic stage affected by the *jas* mutation, we analyzed chromosome behavior during male meiosis in *jas* mutants. Chromosome spreads showed that meiosis in the *jas* mutant progressed normally and was indistinguishable from wild-type meiosis until the end of telophase I. Synapsis was complete and chiasmata as well as bivalents formed (Figure S2A, S2B, S2C, S2G, S2H, S2I). At metaphase II, however, we observed differences to wild-type meiosis. Whereas wild-type chromosomes aligned into two well separated metaphase II plates (Figure S2D), *jas* chromosomes failed to align properly (Figure S2J), likely causing a failure in chromatid separation at the second meiotic division (Figure S2K) and the formation of dyads (Figure S2L) and triads (Figure S2M), in contrast to predominantly occurring tetrad formation in wild-type (Figure S2N).

**Figure 4:**
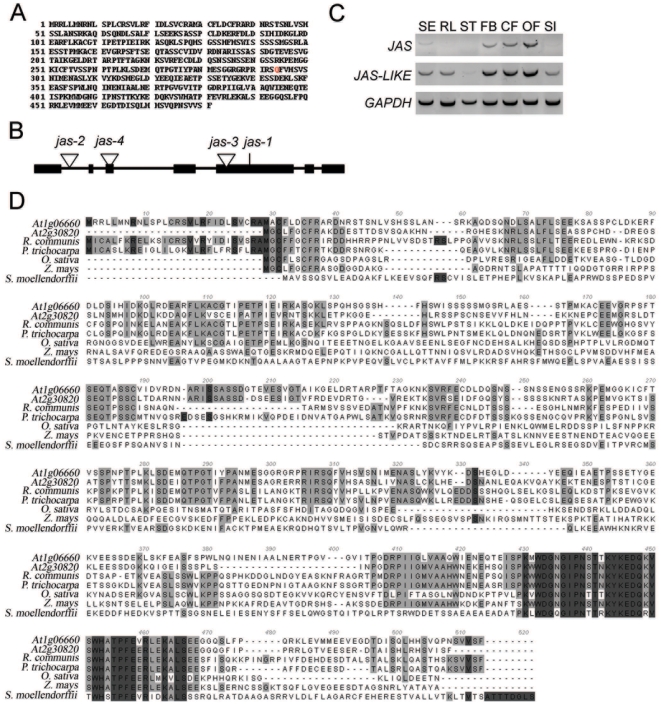
Structure of *JAS* gene and location of mutations. (A) Protein sequence of JAS with site of *jas-1* mutation indicated in red. (B) Exon-intron structure of *JAS* locus, with exons marked by black boxes, introns by black lines. Positions of *jas* alleles are indicated. (C) Expression analysis of *JAS* and *JAS_LIKE* in different plant organs. SE, seedlings; RL, rosette leaves; ST, stem; FB, floral buds (stage 9 flower); CF, closed flower (stage 11 flower); OF, open flower (stage 13 flower); SI, silique. Flower stages were determined as described in [66]. (D) Multiple sequence alignment showing regions of highest sequence conservation among plant JAS proteins. Dark grey and light gray shading symbolizes 100% and over 50% sequence conservation at that position, respectively. Included in the alignment are JAS homologs from dicots *Arabidopsis thaliana* and *Populus trichocarpa*, from monocots *Oryza sativa* and *Zea mays* and from the lycophyte *Selaginella moellendorffii*.

We map-based cloned the *JAS* gene and found the *jas* mutation to cause a premature stop codon in the fifth exon of the *At1g06660* gene, encoding an as yet unknown protein without domains of described functions (Figure 4A and 4B). BLAST searches of *Arabidopsis* proteins revealed a single related protein (At2g30820; JAS-LIKE) with 64% similarity. *JAS* was predominantly expressed during reproductive development, whereas *JAS-LIKE* has a broader expression domain (Figure 4C), suggesting partially redundant functions of both genes. JAS homologs were not identified in animals; however, JAS is conserved throughout the plant kingdom and contains a highly conserved domain at the C-terminus (Figure 4D). We identified three independent T-DNA lines containing insertions in intron 1 (*jas-2*), exon 5 (*jas-3*) and exon 3 (*jas-4*). Mutant alleles *jas-3* and *jas-4* caused dyad and triad formation at comparable frequency as the *jas-1* mutant (Figure 3E), confirming that the identified mutation is indeed the cause of the *jas* phenotype. The frequency of dyad formation in the *jas-2* allele was approximately half that of the other alleles (data not shown), indicating that the *jas-2* mutant allele is not completely penetrant. We also generated and analyzed F1 *jas-1/jas*-3 plants. While plants heterozygous for either the *jas-1* or *jas-3* allele did not produce dyads, we clearly observed dyad formation in F1 plants containing both alleles (Figure 3E). The presence of dyads in multiple *jas* alleles and in plants containing both the *jas-1* and *jas-3* alleles confirms that mutations in the *JAS* gene are indeed the cause of the *jas* phenotype.

### Triploid Seeds Have Increased Expression of FIS Target Genes

**Figure 5:**
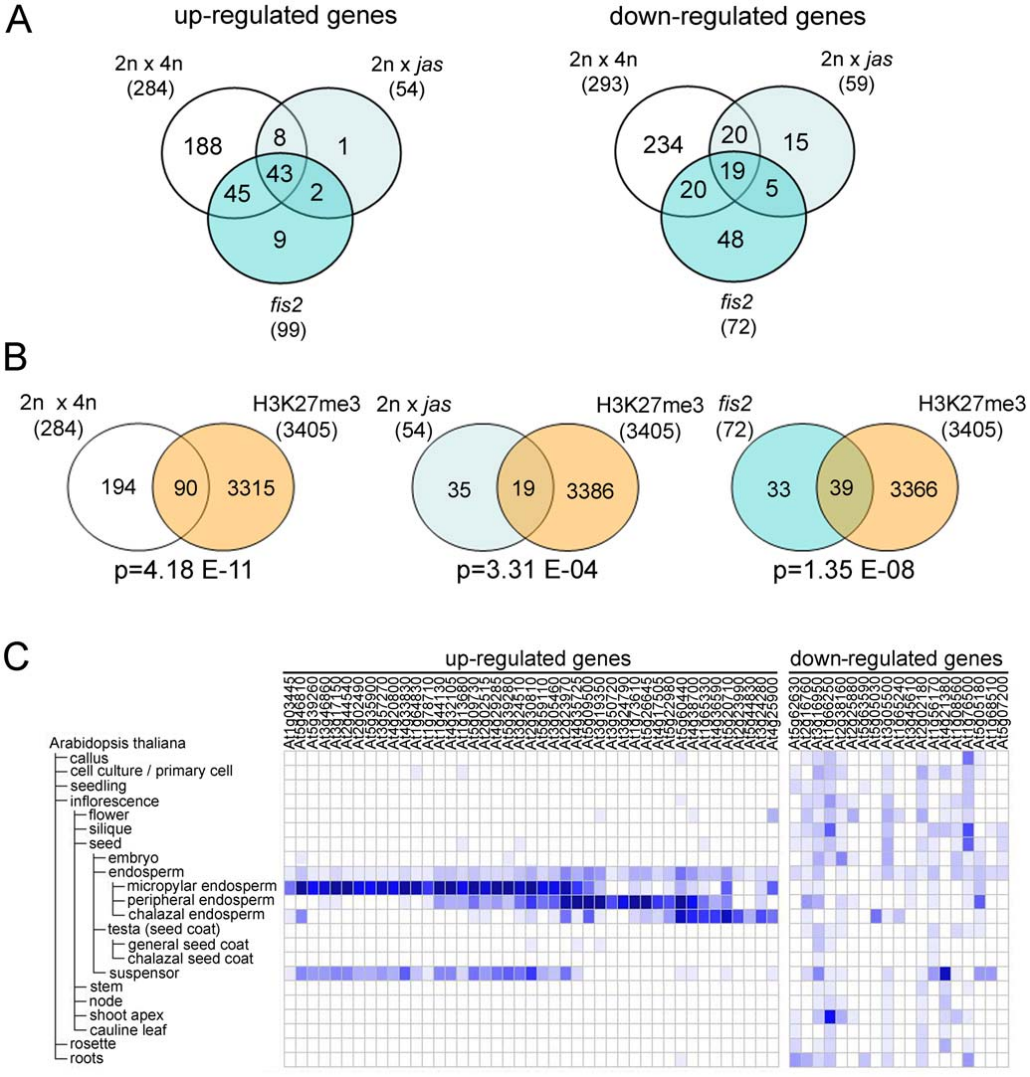
Transcriptome profiles of triploid seeds derived from *jas* and tetraploid pollen parents and *fis2* mutants. (A) Venn diagrams of up-regulated and down-regulated genes in seeds at 6DAP derived from crosses of wild-type plants with pollen from either *jas* plants or tetraploid plants as well as manually self-pollinated *fis2* mutants. Numbers in parenthesis represent total numbers of up-regulated and down-regulated genes in the respective genotypes. (B) Venn diagrams of up-regulated genes in seeds at 6DAP derived from crosses of wild-type plants with pollen from either *jas* plants or tetraploid plants as well as manually self-pollinated *fis2* mutants and genes marked by H3K27me3 [34]. p-values in (A) and (B) are based on the hypergeometric test. (C) Organ-specific expression profiles of up- and down-regulated genes were extracted from publicly available microarray data [64].

*PHE1* is a direct target gene of the FIS PcG complex [31], and we wondered whether increased paternal genome contribution would cause a reactivation of other FIS target genes as well. To investigate this question, we profiled transcriptomes of seeds derived from crosses of wild-type plants with pollen from either *jas* plants or tetraploid plants as well as mutants lacking the FIS subunit FIS2 [33], which were manually self-pollinated. Consistent with the finding that *jas* pollen is diploid, we observed a strong overlap among genes that were deregulated in seeds derived from crosses of wild-type plants with pollen from either *jas* or tetraploid plants (p = 9.11E-99 for up-regulated genes and p = 1.61E-62 for down-regulated genes; Figure 5A and Table S1). The lower number of deregulated genes in seeds of *jas* pollen parents is attributed to the fact that only about half of these seeds are triploid (Table 1), reducing the statistical power to detect small expression changes, whereas all seeds derived from tetraploid pollen parents are triploid. Strikingly, 83% of up-regulated and 41% of down-regulated genes in seeds derived from *jas* pollen parents were also up- or down-regulated in mutants lacking FIS function (p = 7.84E-106 for up-regulated genes and p = 7.56E-49 for down-regulated genes; Figure 5A). Similarly, 89% of up-regulated and 54% of down-regulated genes in *fis2* mutants were as well up- or down-regulated in triploid seeds derived from tetraploid pollen parents (p = 9.38E-164 for up-regulated genes and p = 2.86E-57 for down-regulated genes; Figure 5A). There was a strong linear relation with a slope close to 1 when comparing fold changes of *jas* or *fis2* seeds to fold changes of seeds derived from tetraploid pollen parents (Figure S3, slope parameter = 0.87 and 0.88, respectively). This demonstrates that not only was the set of differentially expressed genes very similar between the analyzed samples, but that also the magnitude of change was very similar in seeds with increased paternal genome contribution and in seeds lacking FIS PcG function. These findings strongly suggest that increased paternal genome contribution causes global deregulation of direct or indirect target genes of the FIS PcG complex. To substantiate these findings, we tested whether genes with altered expression in triploid seeds were enriched for H3K27me3 [34]. Indeed, genes that were up-regulated in triploid seeds or in seeds lacking FIS PcG function were significantly enriched for H3K27me3, supporting the idea that direct FIS PcG target genes are deregulated in triploid seeds (Figure 5B).

Interestingly, genes commonly up-regulated in the three datasets had a preferential expression in the endosperm and were found in all three endosperm domains (micropylar, peripheral and chalazal domains; Figure 5C). These findings suggest that increased paternal genome contribution as well as lack of FIS PcG function predominantly affects endosperm development. Furthermore, these data indicate that FIS PcG function is required to suppress genes in the endosperm that are required during the earlier stages of endosperm development. In contrast, genes that were commonly down-regulated in all three datasets were expressed in various plant organs, suggesting that developmental perturbations in triploid and *fis2* mutant seeds are caused by reduced expression of genes that have a general role during plant development. This idea is supported by the observation that down-regulated genes in triploid seeds are significantly enriched for genes with functions in cellularization and cell cycle control (Table S2), two processes that have a general role during plant development.

**Figure 6:**
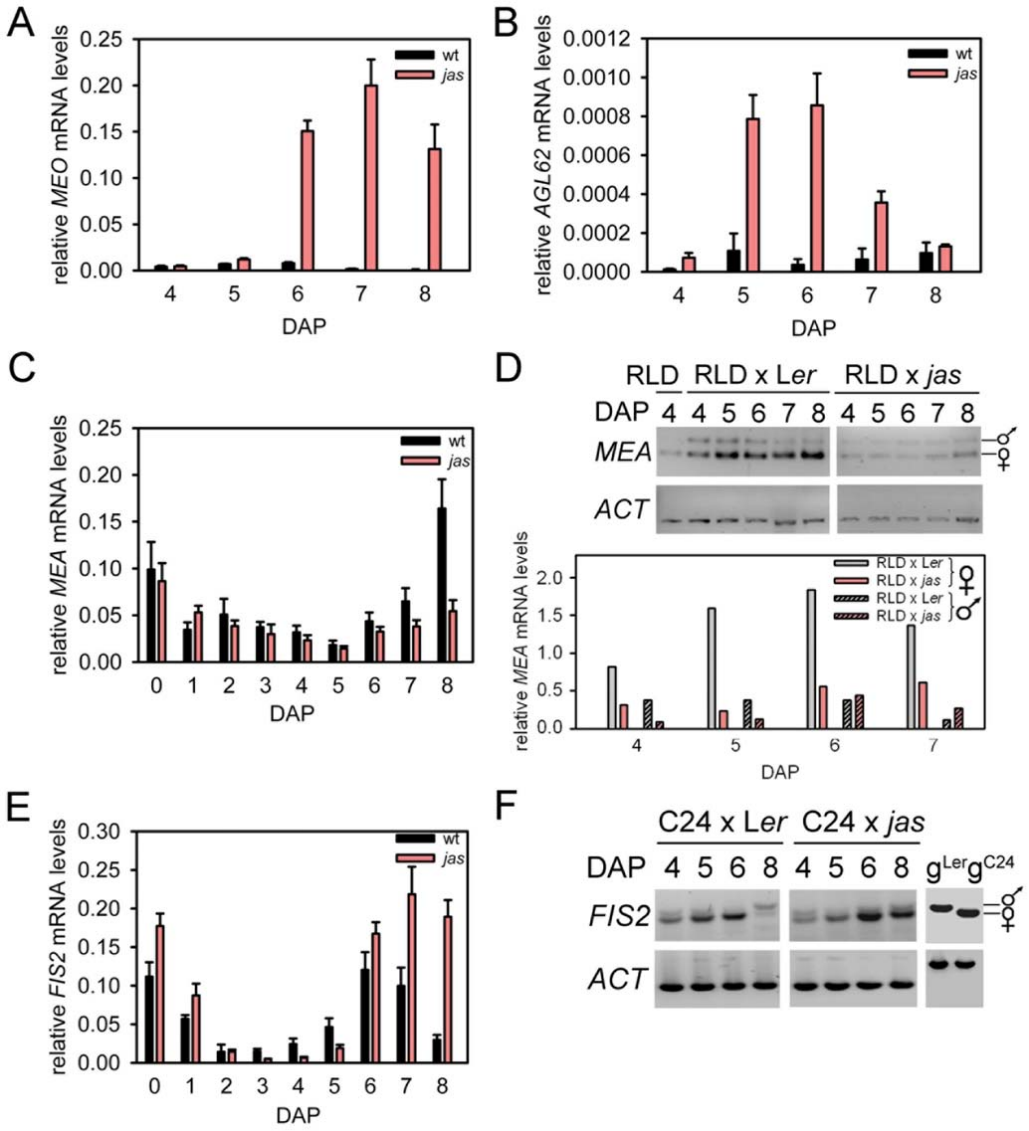
Increased expression of FIS target genes and decreased *MEA* mRNA levels in triploid seeds. Quantitative RT–PCR analysis of *MEO* (A) and *AGL62* (B) expression in wild-type and *jas* seeds. Error bars, s.e.m. (C) Quantitative RT-PCR analysis of *MEA* expression in wild-type and *jas* seeds. Error bars, s.e.m. (D) Allele-specific *MEA* transcript levels were determined after crosses of RLD with wild type (L*er*) or *jas* plants. Quantified results are shown in lower panel. (E) Quantitative RT-PCR analysis of *FIS2* expression in wild-type and *jas* seeds. Error bars, s.e.m. (F) Allele-specific *FIS2* transcript levels were determined after crosses of C24 with wild type (L*er*) or *jas* plants. DAP; days after pollination. wt; wild type.

Among the genes commonly up-regulated in all three datasets we found *PHE1* as well as *MEIDOS* (*MEO*), a gene that we previously identified among genes with deregulated expression in *fis* mutants [31]. Furthermore, we identified the potential FIS target gene *AGL62* [35] as well as several other *AGL* genes (*AGL40*, *AGL36*, *AGL90*; Table S1) with as yet unknown function which, however, were previously shown to interact with PHE1 (AGL40, AGL62) or with interaction partners of PHE1 (AGL36, AGL90) in yeast two-hybrid studies [36]. We tested *MEO* and *AGL62* expression in *jas* seeds at 5–8 DAP and found strongly increased *MEO* and *AGL62* expression levels (Figure 6A and 6B), supporting the results obtained from transcriptome profiling studies. Taken together, these findings strongly support the idea that increased paternal genome contribution causes de-repression of FIS target genes during late seed development.

### Triploid Seeds Have Decreased *MEA* mRNA Levels

Although the FIS subunit *MEA* is expressed until late stages of seed development in embryo and endosperm [28], a role for the FIS complex has only been established to date for female gametophyte and early seed development [37],[38]. Our results suggest that the FIS complex is also required for gene repression in the endosperm during late seed development and that increasing the paternal genome contribution interferes with FIS function. One possible explanation for this finding is that imprinted components of the FIS complex that are only expressed maternally become limited in the endosperm containing an increased paternal genome contribution. We tested this hypothesis by analyzing expression of the paternally imprinted gene *MEA* [28],[30]. We confirmed expression of *MEA* in wild-type seeds 5–8 DAP and, importantly, observed decreased expression levels of *MEA* in seeds of self-fertilized *jas* plants starting at 5–6 DAP (Figure 6C). Because transcript levels were normalized to *ACTIN11* with bi-allelic expression, the increased paternal genome contribution in *jas* is expected to cause an apparent reduction in transcript levels for genes with only maternal expression; thus, the measured changes in relative *MEA* transcript levels were in the expected range. Reduced *MEA* transcript levels were not observed at earlier seed developmental stages, most likely because activation of the paternally contributed genome occurs with a delay of 3–4 days [39] and therefore, increased paternal genome contributions are unlikely to impact on the transcript level of maternally expressed genes.

*MEA* is biallelically expressed in the embryo, but exclusively maternally expressed in the endosperm [28]. As embryo development in triploid *jas* seeds is delayed, we wondered whether decreased transcript levels of *MEA* in *jas* seeds were caused by reduced *MEA* expression in the embryo. Therefore, we tested allele-specific *MEA* transcript levels in seeds derived from crosses of accession RLD with wild-type L*er* or *jas* pollen. We detected strongly decreased transcript levels of the maternal *MEA* allele and only marginally decreased transcript levels of the embryo-derived paternal *MEA* allele in *jas* seeds (Figure 6D). Thus, the observed decreased *MEA* transcript levels in the endosperm were caused by reduced maternal-specific *MEA* transcripts.

*FIS2* encodes a subunit of the FIS complex and is also regulated by genomic imprinting with only the maternal *FIS2* allele being expressed in the endosperm [29],[40]. We also analyzed expression of the *FIS2* gene that like *MEA* had a second expression peak during late seed development (Figure 6E). However, in contrast to reduced *MEA* transcript levels in *jas* seeds, relative *FIS2* transcript levels were increased (Figure 6E). We wondered whether increased *FIS2* expression is caused by activation of the paternal *FIS2* allele. Therefore, we tested allele-specific *FIS2* transcript levels in seeds derived from crosses of accession C24 with wild-type L*er* or *jas* pollen. However, *FIS2* remained imprinted in wild-type and *jas* seeds (Figure 6F), suggesting different regulatory modes of *MEA* and *FIS2* regulation in triploid seeds. Together, our data suggest that developmental changes observed in triploid seeds are caused by lack of sufficient *MEA* rather than *FIS2* expression.

### Increased *MEA* Expression Normalizes Triploid Seed Development

**Figure 7:**
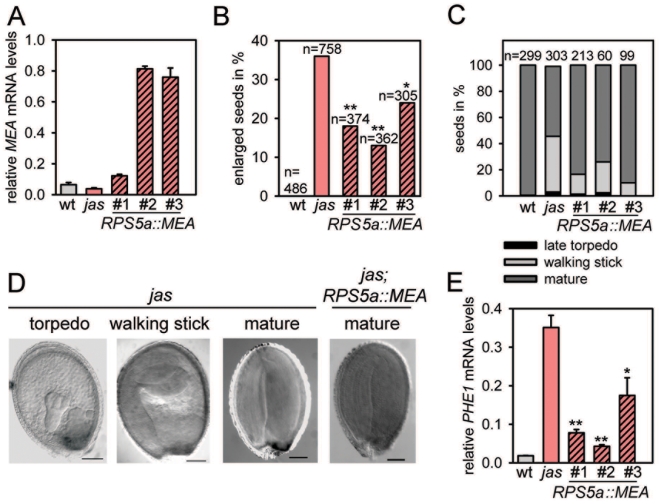
Overexpression of *MEA* partially complements developmental defects of triploid seeds. (A) Quantitative RT–PCR analysis of *MEA* expression in seeds of wild type, *jas* and transgenic lines *jas*; *RPS5::MEA/+* at 7 DAP. Error bars, s.e.m. (B) Number of enlarged seeds in wild-type, *jas* and transgenic lines *jas*; *RPS5::MEA/+*. Significance was determined by Chi-square test comparing normal and enlarged seeds of *jas* and transgenic lines. **P<0.001, *P<0.01, 1 d.f. Numbers above bars indicate numbers of scored seeds per genotype. (C) Distribution of seed developmental stages in *jas* and transgenic lines *jas*; *RPS5::MEA/+* determined at 10 DAP. Numbers above bars indicate numbers of scored seeds per genotype. (D) Progression of embryo development in *jas;RPS5a::MEA* lines. Images correspond to developmental stages quantified in (C). Scale bars, 100 µm. (E) Quantitative RT-PCR analysis of *PHE1* expression in seeds of wild-type, *jas* and transgenic lines *jas*; *RPS5::MEA/+* at 7 DAP. Error bars, s.e.m. Significance was determined by two-tailed Student's t-test. **P<0.001, *P<0.01. DAP; days after pollination. wt; wild type.

If decreased *MEA* transcript levels are causally responsible for developmental aberrations in triploid seeds, increased *MEA* expression levels should normalize development of triploid seeds and partially suppress the *jas* phenotype. To test this hypothesis, we overexpressed *MEA* under control of the *RPS5a* promoter, which is expressed in embryo and endosperm [41]. Complementation of the *mea/+* mutant by a hemizygous *RPS5a::MEA* transgene is expected to result in a reduction of the seed abortion ratio to 25%, as half of the *mea* mutant gametophytes will inherit the transgene. Expression of *MEA* under control of the *RPS5a* promoter in the *mea/+* mutant (*mea/+;RPS5a::MEA/+*) suppressed the *mea* seed abortion phenotype, with 6 out of 14 lines showed seed abortion ratios between 29% (χ^2^ = 2.34<χ^2^_0.01[1]_ = 6.64) and 31% (χ^2^ = 6.52<χ^2^_0.01[1]_ = 6.64), which is close to the expected seed abortion ratio. This suggests that the *RPS5a* promoter is active at the correct stage and at sufficient strength and should be suitable to compensate reduced *MEA* transcript levels in most triploid seeds. We selected three transgenic lines being hemizygous for single locus insertions of the *RPS5a::MEA* construct in the *jas* mutant background that had either wild-type-like or strongly increased *MEA* expression levels (Figure 7A) and assayed the number of enlarged seeds. Consistent with our hypothesis, all lines had significantly less enlarged seeds (Figure 7B, Table S3, Figure S4), with two of the lines having less than half the number of enlarged seeds compared to the *jas* mutant.

We hypothesized that normalization of seed size by *RPS5a::MEA* expression would be associated with a progression of triploid embryo development. We tested this hypothesis by comparing developmental stages of *jas* and *jas;RPS5a::MEA* seeds. Gynoecia of *jas* and *jas;RPS5a::MEA* transgenic lines were manually pollinated and seed development inspected after 10 DAP. Indeed, all three *RPS5a::MEA* containing *jas* lines had reduced numbers of developmentally delayed seeds compared to the *jas* mutant not containing the transgene (Figure 7C), indicating that increased *MEA* expression can normalize seed development in triploid *jas* seeds. In *jas* mutant plants, only segregating wild-type seeds had reached maturity at 10 DAP; whereas triploid *jas* seeds were either in the torpedo or walking stick stage (Figure 7D). We were not able to phenotypically differentiate mature triploid seeds containing the *RPS5a::MEA* transgene from diploid transgene containing seeds (Figure 7D), suggesting that triploid seeds with increased *MEA* expression can complete seed development at similar pace as diploid seeds.

Finally, we asked whether increased *MEA* expression would normalize *PHE1* expression in triploid seeds. Again, consistent with our hypothesis, *PHE1* expression levels were significantly reduced by increasing *MEA* expression in the *jas* mutant (Figure 7E). Together we conclude that reduced *MEA* transcript levels are causally responsible for seed developmental aberrations in triploid *jas* seeds.

## Discussion

Misbalanced expression of imprinted genes has long been implicated as the cause of seed development defects after interploidy crosses [9],[14],[15] Our study provides strong evidence in favor of this hypothesis and demonstrates that *MEA* imprinting is a major origin of developmental defects caused by increased paternal genome contributions. In this work we took advantage of the *jas* mutant that forms unreduced diploid pollen at high frequency, which allowed us to create first generation polyploids and to investigate direct effects of chromosome doubling on seed development. Lack of JAS function causes failure of chromatid segregation during meiosis II, leading to second division nuclear restitution. This mechanism is different to the formation of unreduced pollen by parallel spindles during meiosis II or omission of the second meiotic division, as it occurs in the recently identified *Atps1* and *osd1* mutants, respectively [42],[43]. It also clearly differs from the male defect of the *switch1* (*dyad*) mutant that is defective in prophase I, leading to aberrant segregation of chromatids during the first meiotic division [44]. Thus, the identified *JAS* gene provides molecular insights into a novel mechanism of unreduced pollen formation in plants and will further our understanding of the underlying molecular mechanisms of polyploidy formation.

Similarities of the endosperm phenotype in triploid *jas* seeds and seeds lacking components of the FIS PcG complex let us to propose that FIS function is impaired in seeds with increased paternal genome contribution. We show that FIS genes *MEA* and *FIS2* are expressed at later stages of seed development, concomitantly with the time of endosperm cellularization [45]. Endosperm cellularization in triploid Arabidopsis seeds with paternal excess is delayed or fails completely [18],[19], consistent with increased expression of FIS target genes like *PHE1* and its proposed interaction partner *AGL62* [36], which is implicated to suppress endosperm cellularization [35]. This suggests that the FIS PcG complex has a function during later stages of seed development to suppress inhibitors of endosperm cellularization. We did not observe deregulation of FIS PcG target genes during early development of triploid *jas* seeds, indicating that only this later function of the FIS PcG complex is impaired by increased paternal genome dose in the endosperm. This suggest that the number of accessible FIS target sites increases at the time of endosperm fertilization, consistent with our finding that the paternal *PHE1* allele becomes targeted and silenced by the FIS PcG complex at this time.

*MEA* and *FIS2* are regulated by genomic imprinting and are only maternally expressed [24],[28],[30]. Therefore, we hypothesized that transcript levels of both genes could become limiting in triploid seeds with increased paternal genome contribution. The paternal *MEA* allele is silenced by the MEA containing FIS complex [46]–[48]. Therefore, reduced *MEA* transcript levels in tetraploid *jas* endosperm could potentially cause a breakdown of *MEA* imprinting, leading to a reactivation of the paternal *MEA* allele. However, we show that *MEA* remains imprinted in tetraploid endosperm, suggesting that *MEA* is able to recruit sufficient FIS complexes leading to stable silencing throughout seed development, while many other FIS target genes including *MEO*, *AGL62* and the paternal *PHE1* allele, are not. This implicates that different FIS target genes have different binding affinities for the FIS PcG complex, which is consistent with observations made in Drosophila that dependent on the genomic context, PcG proteins have different binding affinities to their targets [49]. At the *PHE1* locus, different binding affinities of the FIS PcG complex to maternal and paternal alleles might be caused by a differentially methylated region located downstream of the *PHE1* locus that is required for repression of maternal *PHE1* alleles [50].

Although *FIS2* remained imprinted in triploid *jas* seeds, *FIS2* transcript levels were increased compared to wild-type seeds. This suggests that activation of maternal *FIS2* alleles requires transcriptional activators that are induced by increased paternal genome dose in the endosperm. Support for this idea comes from a recent study of Jullien and colleagues [51], who propose the requirement of additional activating factors for *FIS2* expression in the endosperm, based on the finding that lack of DNA methylation does not lead to *FIS2* activation in vegetative tissues.

Increased *MEA* expression normalized triploid seed development and caused triploid embryo development to progress at similar pace like wild-type embryos. About half the number of triploid *jas* seeds were affected by increased *MEA* expression, indicating that either *MEA* expression levels were not sufficiently enhanced in all seeds, or, alternatively, that there are additional factors required to restore normal seed development. Expression of *MEA* under control of its endogenous promoter [25] as well as under control of the *RPS5a* promoter (this study) significantly suppressed abortion of *mea* mutant seeds; however, there was a deviation from the expected seed abortion ratio of 4 to 7%, indicating that indeed transgene-derived *MEA* expression levels are not in all seeds sufficient to restore wild-type seed development.

Interestingly, postygotic lethality of hybrids between *A. thaliana* and *A. arenosa* seems to depend on reduced expression of the *FIS2* and increased expression of AGL family members *AGL62* and *AGL90*, indicating that disturbed FIS complex function might contribute to hybrid seed failure as well [52]. Indeed, increased maternal genome dose strongly suppressed hybrid incompatibility in crosses of tetraploid *A. thaliana* and *A. arenosa* [53], suggesting that increased transcript levels of maternal-specific FIS genes permit normal seed development. Consistently, several other studies reported that there are reciprocal differences in interploidy crossing success, with unreduced eggs being more effective in polyploid formation than unreduced pollen [4], suggesting that increased dosage of PcG complexes is less detrimental for endosperm development than lack of sufficient PcG function. Whether indeed increased maternal genome dose causes repression of FIS PcG target genes will be subject of future investigation.

Maize endosperm is highly dosage sensitive and deviations from the 2∶1 maternal to paternal genome dose will ultimately cause seed abortion [13],[14]. The *MEA* homolog *Mez1* is also imprinted in the maize endosperm [54], suggesting that dosage sensitivity in the endosperm is caused by a conserved mechanism involving imprinted expression of PcG genes. Finally, the *Sfmbt2* PcG gene has recently been shown to be imprinted in trophoblast tissues of mouse embryos [55]. Trophoblast tissues are particularly sensitive to perturbations in genomic imprinting, reflected by dysmorphic trophoblast development in interspecies hybrids [56] and uniparental embryos [57]. Thus, it is possible that imprinting of PcG genes in embryo-nourishing tissues of flowering plants and mammals is an evolutionary conserved system ensuring correct parental genome contributions in the developing progeny.

## Materials and Methods

### Plant Material and Growth Conditions

Plants were grown in a growth room at 70% humidity and daily cycles of 16 h light at 21°C and 8 h darkness at 18°C. The *jas-1* allele was induced in the Landsberg *erecta* (L*er*) accession by ethyl methanesulfonate mutagenesis and harbors a premature stop codon at amino acid position 294 (C-to-T nucleotide substitution at position +1960 of the genomic sequence). Unless otherwise stated, all experiments were performed with the *jas-1* allele. Additional alleles in the Columbia (Col) accession were found in T-DNA insertion libraries: *jas-2* (SALK_083575), *jas-3* (SAIL_813_H03) and *jas-4* (SALK_042866) harboring insertions in intron 1, exon 5, and exon 3, respectively. The *fis2-1* mutant (L*er* accession) has been previously described [33]. Tetraploid lines of L*er* were obtained form the Nottingham Arabidopsis Stock Centre. The *RPS5a::MEA* overexpressing lines were generated by *Agrobacterium tumefaciens* mediated transformation into *jas-1* heterozygous plants and five transgenic lines homozygous for the *jas-1* mutation were analyzed. The *PHE1*::*GUS* line has been described previously [31]. This line was used as the corresponding wild-type control for allele-specific expression analysis. For crosses, designated female partners were emasculated, and the pistils hand-pollinated one day after emasculation. For RNA expression analysis, three siliques were harvested at each of the indicated time points.

### Genetic Screen and Mapping

The *PHE1*::*GUS* line, mutagenized by ethyl methanesulfonate (EMS) treatment, was screened for mutants by selecting M2 plants that showed GUS activity during late stages of seed development. For genetic mapping of the *jas* mutation, we established an F2 mapping population by crossing *jas* with the Col-0 accession. Analyzing 280 *jas* F2 plants using PCR-based polymorphisms, the mutation was located on chromosome 1 in an area of 570 kb between polymorphisms SM104_106,6 and PAI1.2 on BACs T21E18 and F24B9, respectively. Open reading frames within this region were PCR-amplified and analyzed using the SURVEYOR Mutation Detection Kit (Transgenomic). A polymorphism was detected in *At1g06660* (*JAS*) and confirmed by sequencing.

### GUS Expression Analysis and Phenotypic Characterization of Seeds and Pollen

Siliques were harvested for GUS staining at the indicated time points. Staining of seeds to detect GUS activity was done as described previously [31]. Mature pollen nuclei were visualized after staining with 4′,6-diamidino-2 phenylindole (DAPI) as described previously [58]. Buds were harvested for microscopic analysis of tetrad formation and fixed overnight in 3∶1 ethanol∶acetic acid for about 24 h. Buds were then separated and anthers dissected to release pollen into clearing solution (67% chloralhydrate in 8% glycerol) or into DAPI staining solution (100 mM sodium phosphate [pH 7.0], 1 mM EDTA, 0.1% Triton X-100, and 0.4 mg/ml DAPI, high grade; Sigma). Microscopy imaging was performed using a Leica DM 2500 microscope (Leica) with either bright-field or epifluorescence optics. Images were captured using a Leica DFC300 FX digital camera (Leica), exported using Leica Application Suite Version 2.4.0.R1 (Leica Microsystems), and processed using Photoshop 7.0 (Adobe).

### Chromosome Analysis

For chromosome spreads, inflorescences were harvested and fixed in 3∶1 (ethanol∶acetic acid) at −20°C overnight. Flower buds (0.3–0.8 mm) were fixed, equilibrated in citric buffer (10 mM sodium citrate, pH 4.8) and incubated with 1% cytohelicase, 1% pectolyase and 1% cellulase in citric buffer for 3–4 hours at 37°C. Squashes made in 45% acetic acid were air-dried and mounted in antifade containing 4′,6-diamidino-2-phenylindole (DAPI). Slides were analyzed with a Zeiss Axioscope fluorescence microscope (Zeiss, Germany) equipped with a cooled CCD camera (Visitron, Germany). Images were acquired using MetaView software (Universal Imaging Corporation, USA).

### Flow Cytometry

The ploidy levels of leaf cell nuclei were determined by flow cytometry using a PA ploidy analyzer (Partec). Leaves were chopped with a razor blade in CyStain extraction buffer (Partec), filtered through a 30-µm CellTrics filter (Partec) into a sample tube, and stained with CyStain Staining buffer (Partec). Data were collected for approximately 10,000 nuclei per run and were presented on a linear scale.

### Plasmid Constructs

The 1.6 kb upstream sequence of the *RPS5a* translational start was cloned into pB7WG2 vector replacing the 35S promoter. The *MEA* cDNA was cloned into pENTR/D-TOPO (Invitrogen). The *RPS5a::MEA* overexpressing construct was generated by clonase reaction (Invitrogen) between pB7WG2/Rps5a and pENTR/D-TOPO/MEA

### RNA Extraction and qPCR Analysis

RNA extraction and generation of cDNAs were performed using RNAeasy Plant Mini Kit (Qiagen) according to the supplier's instructions. Quantitative PCR was done on an ABI Prism 7700 Sequence Detection System (Applied Biosystems) using SYBR Green PCR master mix (Applied Biosystems) according to the supplier's recommendations. Quantitative RT-PCR was performed using three replicates and results were analyzed as described [59] using *ACTIN11* as a reference gene. Briefly, mean expression values and standard errors for the reference gene as well as for the target genes were determined, taking into consideration the primer efficiency that was determined for each primer pair used. Relative expression values were determined by calculating the ratio of target gene expression and reference gene expression and error bars were derived by error propagation calculation. The primers used in this study are specified in Table S4.

### Allele-Specific Expression Analysis

Primers for *PHE1*, *MEA* and *FIS2* allele specific expression analysis are specified in Table S2. Allele-specific *FIS2* expression analysis was done by crossing C24 and L*er* accessions. The amplified products were digested with *Afl*III and analyzed on a 2.5% agarose gel. Allele-specific *MEA* and *PHE1* expression was analyzed as described previously [27],[28].

### Microarray Analysis

#### Samples, array design, and hybridizations

The three largest flower buds of the inflorescences were emasculated, and the gynoecia were pollinated one day later. Seeds of at least 20 siliques per sample and three independent biological replicates were harvested into 40 µl RNA*later* (Sigma, Buchs, Switzerland) at 6DAP. Glass beads (1.25–1.55 mm) were added, and the samples were ground unfrozen in a Silamat S5 (Ivoclar Vivadent, Ellwangen, Germany). RNA was extracted using the RNAqueous kit (Ambion/Applied Biosystems, Lincoln, CA) combined with Plant RNA Isolation Aid (Ambion/Applied Biosystems) according to manufacturer's instructions. The RNA was subjected to a DNase treatment. cDNA was prepared from total RNA using the WTOvation Pico System (NuGEN Technologies, San Carlos, CA) according to manufacturer's instructions. SPIA amplification was used to prepare single-stranded cDNA from the mRNA starting material. Fragmented and biotin-labeled single-stranded cDNA targets were generated with the FL-Ovation cDNA Biotin Module V2 (NuGEN). Affymetrix Arabidopsis ATH1 GeneChips were used throughout the experiment (Affymetrix, Santa Clara, CA). The exact list of probes present on the arrays can be obtained from the manufacturer's website (http://www.affymetrix.com). Analysis was based upon annotations compiled by TAIR (www.arabidopsis.org, version 2007-5-2). Data were deposited into the ArrayExpress database (Accession number E-MEXP-2316).

#### Bioinformatic analysis

All data processing was performed using the statistic package R (version 2.8.1.) that is freely available at http://www.r-project.org/ [60]. Signal values were derived from Affymetrix CEL files using GCRMA. Quality control was done using the *affyQCReport* package in R. In addition, we calculated coefficients of variation (cv) between replicates as a quantitative measure of data quality and consistency between replicates as described previously [31]. Median cv values for triplicate array sets were between 1.4 and 1.8% demonstrating the high quality of the data. Because in *jas* and *fis2* only about 50% of the seeds are mutant, observed fold changes (FC_obs_) were smaller than real FC (FC_real_) in mutant seeds. FC_real_ values were estimated using equation FC_real_ = 2×FC_obs_−1.

Differentially expressed genes were identified using the *limma* package in R [61]. Multiple-testing correction was done using the q-value method [62]. Probe sets were called significantly differentially expressed when q<0.075. To compensate for the increased statistical power of the 2n×4n cross, where not only 50% but all seeds are affected, q<0.05 was required in this case. To enrich for biologically relevant changes, only probe sets with FC_real_>2 or FC_real_<−2 were selected. Differentially expressed genes were grouped into functional gene ontology categories (obtained from www.arabidopsis.org). Data for H3K27me3 target loci were from [34]. The significance of enrichment was estimated based on the hypergeometric test and multiple-testing correction according to [63] with a critical p-value of 0.01. Analysis of tissue-specificity of differentially expressed genes was performed in Genevestigator [64].

## Supporting Information

Figure S1 Paternal transmission of the *jas* mutation causes changes in seed size. (A) Seed size histogram of seeds derived from self-fertilized wild-type plants and self-fertilized *jas* mutants. (B) Seed size histogram of seeds derived from self-fertilized wild-type plants and *jas* mutants fertilized with wild-type pollen. Mean seed size of corresponding wild-type seeds was set to one. For each sample 151 seeds were analyzed.
(0.38 MB TIF)

Figure S2 Meiosis I is normal but meiosis II is defective in *jas* mutants. (A–F) Wild-type meiotic chromosome spreads. (A) Pachytene. (B) Metaphase I. (C) Anaphase I. (D) Metaphase II. (E) Anaphase II (F) Telophase II. (G–N) *jas* meiosis. (G) Pachytene. (H) Metaphase I. (I) Anaphase I. (J) Metaphase II. (K) Anaphase II. (L) Dyad. (M) Triad. (N) Tetrad. Tetrad. Scale bar, 10 µm.
(0.53 MB TIF)

Figure S3 Comparison of fold changes between 2n×*jas*, *fis2* and 2n×4n samples. (A) Fold change comparison between 2n×*jas* and 2n×4n samples. (B) Fold change comparison between *fis2* and 2n×4n samples.
(0.24 MB TIF)

Figure S4 Overexpression of *MEA* reduces triploid seed size. (A) Seed size histogram of seeds derived from self-fertilized wild-type plants and self-fertilized *jas* mutants. (B–D) Seed size histograms of seeds derived from self-fertilized *jas* mutants and self-fertilized *jas;RPS5a::MEA/+* transgenic lines. (B) Line1. (C) Line2. (D) Line3. Mean seed size of corresponding wild-type seeds was set to one. For each sample 151 seeds were analyzed.
(0.54 MB TIF)

Table S1 Deregulated genes in triploid *jas* seeds and seeds derived from crosses of 2n×4n plants.
(0.14 MB XLS)

Table S2 Functional characterization of genes deregulated in seeds at 6DAP derived from crosses of wild-type plants with pollen from either *jas* plants or tetraploid plants as well as manually self-pollinated *fis2* mutants.
(0.08 MB PDF)

Table S3 Seed phenotypes of wild-type, *jas*, and transgenic lines *jas;RPS5a::MEA/+*. Green or dry siliques were opened and the seeds classified as normal, enlarged, aborted, or unfertilized ovules. n (seeds), number of seeds scored. P values were determined by Chi-square test comparing normal and enlarged seeds of *jas* and transgenic lines. N.a.; not applicable.
(0.06 MB PDF)

Table S4 Primers used in this study.
(0.05 MB PDF)
