# Supplementary figures and images for: Imprinting of the Polycomb Group Gene MEDEA Serves as a Ploidy Sensor in Arabidopsis
Source: PLoS Genet. 2009 Sep 25;5(9):e1000663. doi: 10.1371/journal.pgen.1000663 (PMC2738949; doi:10.1371/journal.pgen.1000663)

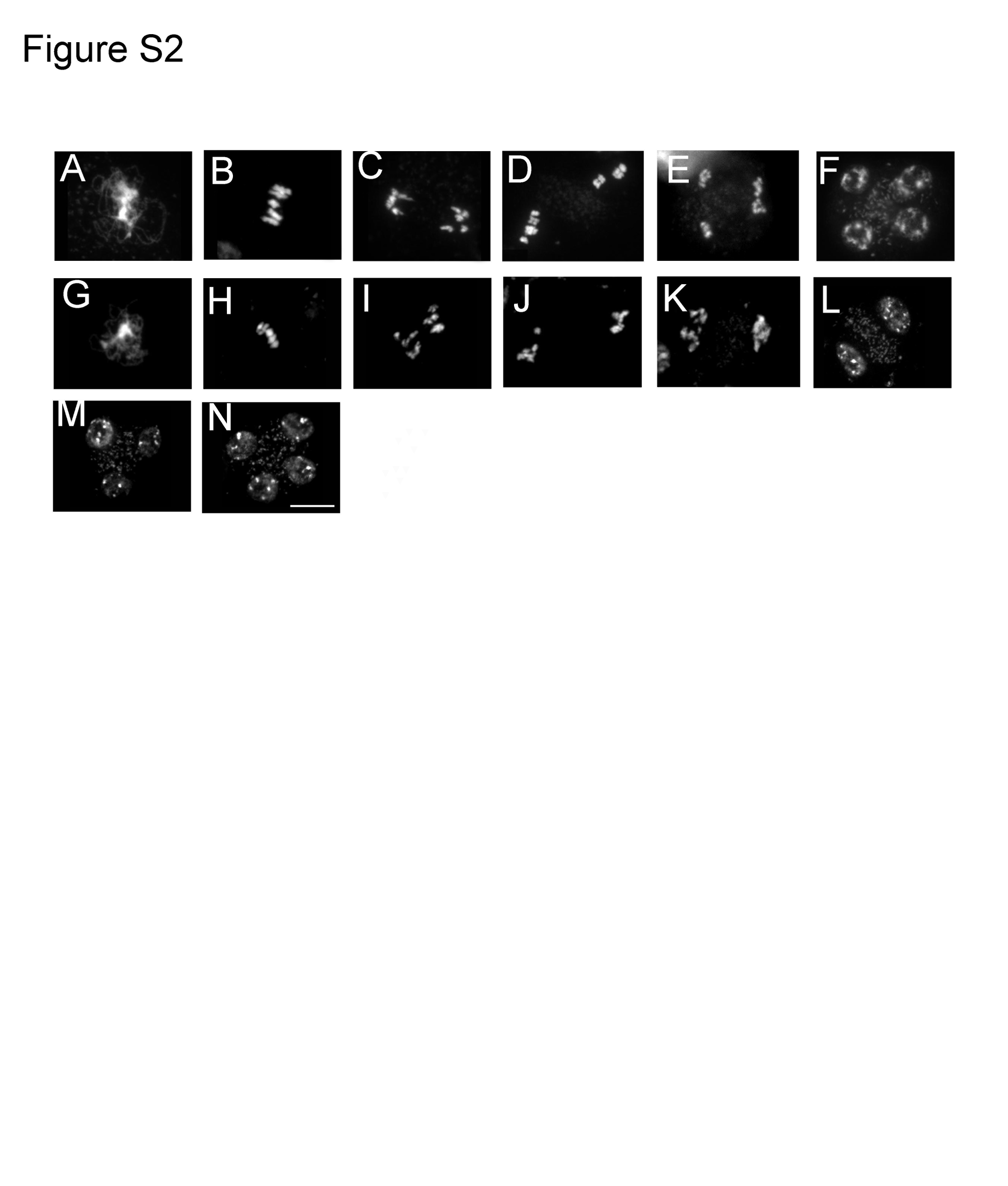

Supplement: Figure S2 — Meiosis I is normal but meiosis II is defective in jas mutants. (A–F) Wild-type meiotic chromosome spreads. (A) Pachytene. (B) Metaphase I. (C) Anaphase I. (D) Metaphase II. (E) Anaphase II (F) Telophase II. (G–N) jas meiosis. (G) Pachytene. (H) Metaphase I. (I) Anaphase I. (J) Metaphase II. (K) Anaphase II. (L) Dyad. (M) Triad. (N) Tetrad. Tetrad. Scale bar, 10 µm. (0.53 MB TIF) [file pgen.1000663.s002.tif]

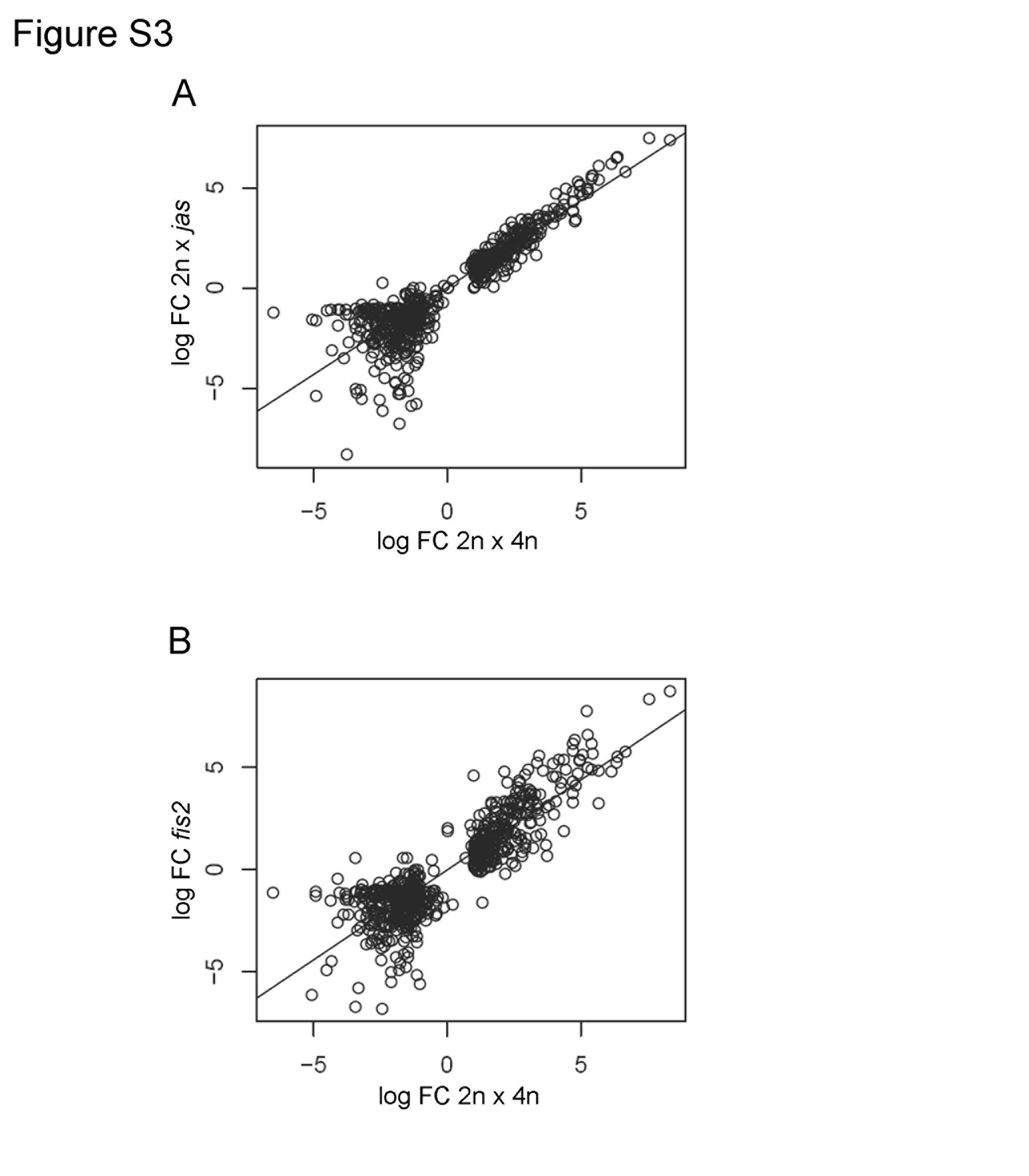

Supplement: Figure S3 — Comparison of fold changes between 2n×jas, fis2 and 2n×4n samples. (A) Fold change comparison between 2n×jas and 2n×4n samples. (B) Fold change comparison between fis2 and 2n×4n samples. (0.24 MB TIF) [file pgen.1000663.s003.tif]
