# Supplementary material for: Imprinting of the Polycomb Group Gene MEDEA Serves as a Ploidy Sensor in Arabidopsis
Source: PLoS Genet. 2009 Sep 25;5(9):e1000663. doi: 10.1371/journal.pgen.1000663 (PMC2738949; doi:10.1371/journal.pgen.1000663)
Supplement: Table S2 — Functional characterization of genes deregulated in seeds at 6DAP derived from crosses of wild-type plants with pollen from either jas plants or tetraploid plants as well as manually self-pollinated fis2 mutants. (0.08 MB PDF) [file pgen.1000663.s006.pdf]

Table S2

GO category	Found frequency	Expected frequency	p value
**2n x 4n upregulated genes**			
endomembrane system	80	40.80699	1.03E-08
hydrolase activity, hydrolyzing O-glycosyl compounds	15	2.486538	1.21E-07
carbohydrate metabolic process	17	4.99832	8.48E-05
carboxylesterase activity	8	1.628241	0.001015
**2n x *jas* upregulated genes**			
endomembrane system	28	7.632963	1.89E-10
hydrolase activity, hydrolyzing O-glycosyl compounds	6	0.465108	1.10E-05
carbohydrate metabolic process	7	0.934938	0.000107
***fis2 x fis2* upregulated genes**			
endomembrane system	40	13.94484	5.52E-10
hydrolase activity, hydrolyzing O-glycosyl compounds	5	0.849716	0.005737
carbohydrate metabolic process	7	1.708059	0.00828
**2n x 4n downregulated genes**			
regulation of cell cycle	9	0.803178207	1.79E-07
cyclin-dependent protein kinase regulator activity	6	0.302837684	3.47E-07
acyl carrier activity	3	0.171169126	0.000490423
microtubule motor activity	6	0.987514188	0.001466402
phragmoplast	6	1.000681044	0.001586177
polarity specification of adaxial/abaxial axis	3	0.263337117	0.002903381
microtubule associated complex	5	0.829511918	0.004009816
