# Supplementary material for: Imprinting of the Polycomb Group Gene MEDEA Serves as a Ploidy Sensor in Arabidopsis
Source: PLoS Genet. 2009 Sep 25;5(9):e1000663. doi: 10.1371/journal.pgen.1000663 (PMC2738949; doi:10.1371/journal.pgen.1000663)
Supplement: Table S3 — Seed phenotypes of wild-type, jas, and transgenic lines jas;RPS5a::MEA/+. Green or dry siliques were opened and the seeds classified as normal, enlarged, aborted, or unfertilized ovules. n (seeds), number of seeds scored. P values were determined by Chi-square test comparing normal and enlarged seeds of jas and transgenic lines. N.a.; not applicable. (0.06 MB PDF) [file pgen.1000663.s007.pdf]

Table S3

Genotype	normal	enlarged	aborted	unfertilized	n	X^2 (1d.f.)	P
wild type	89%	0%	3%	8%	486	n.a.	
jas	51%	36%	2%	11%	758	n.a.	
jas;RPS5a::MEA #1	66%	18%	3%	13%	374	42,6160	<0.001
jas;RPS5a::MEA #2	69%	13%	4%	14%	362	61,9122	<0.001
jas;RPS5a::MEA #3	52 %	24%	3%	21%	305	7,3579	<0.01
