# Supplementary material for: Imprinting of the Polycomb Group Gene MEDEA Serves as a Ploidy Sensor in Arabidopsis
Source: PLoS Genet. 2009 Sep 25;5(9):e1000663. doi: 10.1371/journal.pgen.1000663 (PMC2738949; doi:10.1371/journal.pgen.1000663)
Supplement: Table S4 — Primers used in this study. (0.05 MB PDF) [file pgen.1000663.s008.pdf]

Table S4

PHE1 qPCR primers	TCCAACACCGAAAACTCCAT, CGCATGTGCGGTCATCC
MEO qPCR primers	GTGGCTCGTAAGATGCAGATG, AGCGAGGATCTTTCCAGTGAC
AGL62 qPCR primers	CCTCCTCACCAACACAACAA, ACCTTTGAACCCCTCGAGTT
MEA qPCR primers	GGTGAGGCACTAGAATTGAGCAGT, CCATAGTCCTGCCCAACCG
FIS2 qPCR primers	GATTGTTGGCATTAGCAGCA, CGATTGGTGGTGGAGAATGT
ACT11 qPCR primers	GGAACAGTGTGACTCACACCATC, AAGCTGTTCTTTCCCTCTACGC
PHE1 allele specific primers	CGCATGTGCGGTCATCC, CGTCTCTTGATCCACCATCTTCTTGGTCC
FIS2 allele specific primers	GAACCTAAGGTGCGaCGTG, TCAGCTATTGCTGGTTGAGTCT
MEA allele specific primers	GACCTAACTGCTACGCCAAG, AAGGACTGCTTGAATTGCTGCTTCTCCTCGGATC
ACT11 allele specific primers	AACTTTCAACACTCCTGCCATG, CTGCAAGGTCCAAACGCAGA
JAS RT-PCR primers	TCTCGTTTGGCTGAATCCTC, TCAGCCTCTATCTGCTCTTCG
JAS-LIKE RT-PCR primers	CGATCTTCCCCTTCCTGTAA, TTCGCATTTGAATCTTCGTG
GAPDH RT-PCR primers	ATGGCTTCGGTTACTTTCTCTGTC, TTCTTGGCACCAGCTTCAAT
